# Supplementary material for: Diversity-dependent speciation and extinction in hominins
Source: Nat Ecol Evol. 2024 Apr 17;8(6):1180–90. doi: 10.1038/s41559-024-02390-z (PMC11166571; doi:10.1038/s41559-024-02390-z)
Supplement: Supplementary file 1 — Supplementary Tables 1–3. [file 41559_2024_2390_MOESM1_ESM.pdf]

nature ecology & evolution

Article https://doi.org/10.1038/s41559-024-02390-z

Diversity-dependent speciation and extinction in hominins

In the format provided by the authors and unedited

Supplementary Materials

Supplementary Table 1: Broadest occurrence level PyRate outputs

		**Mean of posterior distribution of correlation parameter**	**95% HPD interval**	**% of posterior distribution greater or lesser than 0**
Speciation				
Whole clade	Time-based preservation variability	-0.47	-3.51, 0.45	**79.8%**
	Within-lifetime preservation variability	-0.53	-1.96, 0.53	**82.2%**
Non-*Homo*	Time-based preservation variability	0.00	-2.90, 2.40	N/A
	Within-lifetime preservation variability	-0.27	-1.42, 0.66	62.3%
Homo	Time-based preservation variability	0.53	-2.27, 4.00	62.5%
	Within-lifetime preservation variability	2.12	-4.19, 5.95	**84.2%**
Extinction				
Whole clade	Time-based preservation variability	0.21	-1.74, 4.61	64.6%
	Within-lifetime preservation variability	0.16	-0.74, 1.30	60.2%
Non-*Homo*	Time-based preservation variability	0.01	-0.82, 0.95	52.3%
	Within-lifetime preservation variability	0.19	-0.77, 1.31	68.6%
Homo	Time-based preservation variability	**-5.60**	**-9.12, -1.67**	**100%**
	Within-lifetime preservation variability	**-7.92**	**-10.68, -4.97**	**100%**

95% highest posterior density (HPD) interval in bold if it does not overlap with 0. % of posterior distribution greater or lesser than 0 (depending on direction of mean of the posterior distribution) in bold if over 75%. Mean in bold if both 95% HPD interval does not overlap with 0 and % of posterior distribution is in the same direction indicated by the mean is >75%.

Supplementary Table 2: Estimated times of origination and extinction for the broadest occurrence level PyRate models

**Species**	**Within-lifetime variability**		**Time-based variability**	
	Speciation (Ma)	Extinction (Ma)	Speciation (Ma)	Extinction (Ma)
Australopithecus afarensis	3.94 [3.66, 4.27]	2.30 [1.91, 2.89]	3.74 [3.60, 3.99]	2.45 [2.18, 2.91]
Australopithecus africanus	4.09 [3.50, 4.80]	1.64 [0.85, 2.27]	3.79 [3.41, 4.34]	2.10 [1.79, 2.38]
Australopithecus anamensis	4.56 [4.30, 4.83]	3.41 [3.20, 3.57]	4.43 [4.20, 4.70]	3.47 [3.22, 3.60]
Australopithecus bahrelghazali	3.22 [3.02, 3.50]	3.18 [2.98, 3.48]	3.34 [3.02, 3.79]	3.08 [2.72, 3.48]
Australopithecus deyiremeda	3.41 [3.31, 3.78]	3.37 [3.27, 3.50]	3.53 [3.31, 3.89]	3.26 [2.93, 3.50]
Australopithecus garhi	2.51 [2.50, 2.55]	2.49 [2.45, 2.50]	2.57 [2.50, 2.74]	2.44 [2.26, 2.50]
Australopithecus sediba	2.07 [1.90, 2.15]	2.03 [1.88, 2.11]	2.16 [1.90, 2.48]	1.98 [1.80, 2.11]
Homo erectus sensu lato	2.38	0.00	2.14	0.01

	[2.23, 2.54]	[0.00, 0.01]	[2.01, 2.36]	[0.00, 0.01]
Homo floresiensis	1.25<br>[0.82, 1.80]	0.02<br>[0.00, 0.05]	1.19<br>[0.75, 1.94]	0.04<br>[0.00, 0.09]
Homo habilis	2.83<br>[2.56, 3.12]	0.97<br>[0.68, 1.23]	2.55<br>[2.38, 2.80]	1.23<br>[1.14, 1.35]
Homo heidelbergensis	1.33<br>[0.92, 1.73]	0.02<br>[0.00, 0.05]	1.34<br>[0.89, 1.84]	0.08<br>[0.02, 0.13]
Homo neanderthalensis	0.93<br>[0.39, 1.70]	0.00<br>[0.00, 0.01]	0.94<br>[0.39, 1.70]	0.01<br>[0.00, 0.01]
Homo rudolfensis	2.34<br>[2.00, 2.92]	1.27<br>[0.65, 1.77]	2.24<br>[1.95, 2.79]	1.43<br>[1.11, 1.81]
Homo sapiens	0.32<br>[0.28, 0.36]	0	0.32<br>[0.28, 0.36]	0
Paranthropus aethiopicus	3.29<br>[3.00, 3.57]	2.10<br>[1.81, 2.39]	3.24<br>[2.93, 3.56]	2.30<br>[2.12, 2.50]
Paranthropus boisei	3.41<br>[2.94, 3.82]	0.64<br>[0.25, 1.00]	3.34<br>[2.83, 3.80]	1.30<br>[1.11, 1.43]
Paranthropus robustus	2.19<br>[1.88, 2.59]	1.28<br>[0.81, 1.55]	2.19<br>[1.82, 2.73]	1.39<br>[1.10, 1.60]

Numbers in brackets represent 95% credible intervals.

Supplementary Table 3: Output from phylogenetic generalised least squares regression

We ran phylogenetic generalized least squares regressions across the Parins-Fukuchi et al.[1] phylogeny using the *nlme* package[2] in R to ask whether there are differences between *Homo* and non-*Homo* (*Australopithecus* and *Paranthropus*) in the relationship between speciation rates ('DR') and previous clade-wide diversity ('diversity' - species diversity 500k years before the tip height). The phylogenetic correlation structure of residual error in the phylogenetic GLS was accounted for in the *nlme* "correlation" argument. The model assumed a Brownian motion model for residual error structure, following previous work on regressions including speciation rates. Non-contemporaneity of tips was represented in the *nlme* argument "weights".

Model: DR ~ Diversity × Clade

	**Estimate**	**95% CI**	**p (two-sided)**
**Intercept**	-1.5159	(-3.3057, 0.2740)	0.1251
**Diversity**	0.3083	(0.1412, 0.4756)	**0.0041 ***
**Non-Homo**	3.0975	(0.6329, 5.5620)	**0.0315 ***
**Diversity × Non-Homo**	-0.4462	(-0.7560, -0.1365)	**0.0166 ***

Residual standard error: 0.5399581
Degrees of freedom: 15 total; 11 residual

References

1. Parins-Fukuchi, C., Greiner, E., MacLatchy, L. M. & Fisher, D. C. Phylogeny, ancestors, and anagenesis in the hominin fossil record. *Paleobiology* **45**, 378–393 (2019).
2. Pinheiro, J., Bates, D., DebRoy, S., Sarkar, D. & R Core Team. nlme: Linear and Nonlinear Mixed Effects Models. (2020).
