# Supplementary material for: Diversity-dependent speciation and extinction in hominins
Source: Nat Ecol Evol. 2024 Apr 17;8(6):1180–90. doi: 10.1038/s41559-024-02390-z (PMC11166571; doi:10.1038/s41559-024-02390-z)
Supplement: Supplementary file 2 — Reporting Summary [file 41559_2024_2390_MOESM2_ESM.pdf]

nature portfolio

Corresponding author(s): L A van Holstein

Last updated by author(s): 5 January 2024

Reporting Summary

Nature Portfolio wishes to improve the reproducibility of the work that we publish. This form provides structure for consistency and transparency in reporting. For further information on Nature Portfolio policies, see our Editorial Policies and the Editorial Policy Checklist.

Statistics

For all statistical analyses, confirm that the following items are present in the figure legend, table legend, main text, or Methods section.

n/a	Confirmed	
☐	☒	The exact sample size (*n*) for each experimental group/condition, given as a discrete number and unit of measurement
☒	☐	A statement on whether measurements were taken from distinct samples or whether the same sample was measured repeatedly
☐	☒	The statistical test(s) used AND whether they are one- or two-sided<br>*Only common tests should be described solely by name; describe more complex techniques in the Methods section.*
☐	☒	A description of all covariates tested
☐	☒	A description of any assumptions or corrections, such as tests of normality and adjustment for multiple comparisons
☐	☒	A full description of the statistical parameters including central tendency (e.g. means) or other basic estimates (e.g. regression coefficient) AND variation (e.g. standard deviation) or associated estimates of uncertainty (e.g. confidence intervals)
☐	☒	For null hypothesis testing, the test statistic (e.g. *F*, *t*, *r*) with confidence intervals, effect sizes, degrees of freedom and *P* value noted<br>*Give P values as exact values whenever suitable.*
☐	☒	For Bayesian analysis, information on the choice of priors and Markov chain Monte Carlo settings
☒	☐	For hierarchical and complex designs, identification of the appropriate level for tests and full reporting of outcomes
☒	☐	Estimates of effect sizes (e.g. Cohen's *d*, Pearson's *r*), indicating how they were calculated

Our web collection on statistics for biologists contains articles on many of the points above.

Software and code

Policy information about availability of computer code

Data collection

a) Occurrence data and first- and last appearance dates
Fossil occurrence data were obtained from (1) the Paleobiology Database, using the taxon search for 'hominin', (2) the NOW Database, and (3) the ROCEEH ROAD Database, in November 2023. Taxonomy of the Paleobiology Database occurrence data was checked for spelling errors using the PyRate "check_names" function52, and manually for synonyms. Occurrence data from the three databases were merged. Duplicates were identified manually, and record with most up-to-date age estimates retained. If occurrences did not have a specified accession number, duplicates were identified based on location (geological formation and/or member, latitude and longitude) in combination with inspecting specified source publications (if available). To account for differences in the three databases' approach to defining occurrence localities (e.g., Australopithecus afarensis at Laetoli comprises two entries in the Paleobiology Database, both of which are composites of >2 find spots, whilst all find spots are separate entries in the NOW database), we took a hierarchical approach to recording occurrences, recording 'Site complex' (e.g., the Woranso-Mille paleoanthropological research area), 'Site' (e.g., Taung), 'Subsite' (e.g., localities or surface find spots within a 'site'; subsite 'type' was also recorded), 'Formation' (e.g., Koobi Fora), 'Stratigraphic unit' (e.g., Member 4). Not all occurrences have information for all variables: for example, the Mauer site is not part of a larger 'Site complex'. We supplemented and updated the merged database with occurrence information obtained from literature reviews of papers published after 2016, and cross-checked our database with occurrence information supplied in published overviews of research where available.

Species' published first appearance dates (FADs) and last appearance dates (LADs), which are conventionally taken as speciation and extinction 'times', were taken from Wood & Boyle and supplemented with dates of more recently published species in the manner described in van Holstein & Foley.

b) Phylogeny
We used the phylogeny with the best Akaike information criterion score from Parins-Fukuchi et al. In contrast to other hominin phylogenies,

this phylogeny combines probabilistic models of morphological evolution and fossil preservation to recover anagenetic relationships between hominin species. It therefore uniquely recovers ancestor-descendant relationships that are (i) likely more realistic than those on phylogenies that do not incorporate them, and (ii) broadly accepted based on morphological evidence alone (for example, between Australopithecus anamensis and Australopithecus afarensis62).

c) Data from phylogeny: speciation rate and previous diversity for each tip
Jetz et al.'s tip DR, which calculates tip-specific speciation rate, was calculated for every tip.
For each tip, the number of extant species at 500k years before the tip was obtained using the "getExtant" function in the phytools package.

Data analysis

a) Analyses based on speciation and extinction times: Do speciation and extinction times correlate with species diversity?
We ran birth-death models, with diversity as predictor, in a validated Bayesian framework on five datasets with estimated times of species origination and extinction. The first dataset was based on the conservative FADs and LADs estimated by Wood and Boyle, with additions from van Holstein and Foley and thus incorporates no variability in fossil preservation rates. The subsequent four datasets were based on our new hominin occurrence database. From these data, we created four new sets of estimated times of speciation and extinction with two sets of explicit fossil preservation rate priors and two operational definitions of localities (at the finest-grained occurrence level available [n=385 occurrences], and at the broadest occurrence level [i.e., in which all occurrences at a site complex were merged into a single occurrence; n=267 occurrences]). As there are no differences in the direction of inferred relationships between these datasets, we report results for both models of preservation from the most fine-grained occurrence level, and results for broadest occurrence level are provided Supplementary Table 1.

In the first dataset, we modelled fossil preservation as a function of a time-variable Poisson process. Preservation rates were allowed to vary every 1 million years. In the second dataset, we allowed fossil preservation to vary over the course of a species' lifespan by modelling it with a homogeneous Poisson process of preservation (NHPP). This allowed us to take into account that fossils are less likely to form at the start and end of a species' lifespan, as the number of individuals belonging to a species is low. In both datasets, fossil preservation was also allowed to vary between lineages by incorporating a Gamma model of rate heterogeneity. We generated ten replicates of estimated times of species origination and extinction for both preservation regimes using the Reversible Jump MCMC algorithm in the python programme PyRate to incorporate dating uncertainty into the results. All analyses described below were then performed on the 10 replicates, and results were joined into a single posterior sample.

We generated lineage-through-time estimates for all three datasets in Pyrate. We then applied the PyRate exponential birth-death model, with clade-wide lineage-through-time estimates as the predictor, to the estimated speciation and extinction times of Homo and non-Homo species. To compare these results with the pattern across the whole clade, we ran an exponential diversity-dependent birth-death model, in which the whole clade's own diversity is used as the predictor variable. Because the sample size is inevitably relatively small, we ran each model for 2,000,000 iterations, sampling every 1,000 iterations.
b) Analyses based on phylogeny: Is variation in speciation rate predicted by species diversity?
These analyses were performed in R 4.0167. To explore the relationship between speciation and diversity—and in particular, the difference between Homo and non-Homo species further, we ran phylogenetic generalized least squares (GLS) regressions to ask whether there are differences between Homo and non-Homo in the relationship between speciation rates and previous clade-wide diversity. The phylogenetic correlation structure of residual error in the phylogenetic GLS was accounted for in the nlme "correlation" argument. The model assumed a Brownian motion model for residual error structure, following previous work on regressions including speciation rates . Non-contemporaneity of tips was represented in the nlme argument "weights".

To test the ability of the phylogeny-based approach described above to correctly distinguish between diversity-dependent and non-diversity dependent speciation, we simulated 1000 phylogenies under a constant-rate birth-death process using the "pbtree" function in the phytools package64, preserving extinct tips, and repeated the analyses described above for equation (1) to estimate how often diversity-dependent speciation is erroneously inferred across non-ultrametric trees generated under a non-diversity-dependent process. We then generated 1000 phylogenies simulated under a diversity-dependent regime using the "ddsim" function in the DDD package. These phylogenies were simulated with birth and death rates and carrying capacities randomly drawn from a normal distribution with means that produced trees with similar tip numbers to the Parins-Fukuchi et al.58 phylogeny in a trial run, and with a maximum tree height of 7, so as to produce similarly small phylogenies to the Parins-Fukuchi et al.58 tree. We then randomly removed up to 40% of tips and repeated the analyses to investigate the sensitivity of results to incomplete sampling.

We also tested the sensitivity of the results of phylogeny-based analyses using equation (2) to the increased probability of species discovery towards the present, which could have resulted in the underrepresentation of non-Homo species relative to the younger Homo species included in the analyses. To do so, we generated 4000 phylogenies with up to +50% non-Homo species added in random locations and with random tip heights to the original Parins-Fukuchi et al.58 phylogeny using the "bind_tip" function in the phytools package. We repeated the analyses described above for equation (2) and calculated the proportion of trees across which the original results were maintained.

For manuscripts utilizing custom algorithms or software that are central to the research but not yet described in published literature, software must be made available to editors and reviewers. We strongly encourage code deposition in a community repository (e.g. GitHub). See the Nature Portfolio guidelines for submitting code & software for further information.

Data

Policy information about availability of data

All manuscripts must include a data availability statement. This statement should provide the following information, where applicable:

- Accession codes, unique identifiers, or web links for publicly available datasets
- A description of any restrictions on data availability
- For clinical datasets or third party data, please ensure that the statement adheres to our policy

All data and code will be made available on FigShare upon publication.

Research involving human participants, their data, or biological material

Policy information about studies with human participants or human data. See also policy information about sex, gender (identity/presentation), and sexual orientation and race, ethnicity and racism.

Reporting on sex and gender	N/A
Reporting on race, ethnicity, or other socially relevant groupings	N/A
Population characteristics	N/A
Recruitment	N/A
Ethics oversight	N/A

Note that full information on the approval of the study protocol must also be provided in the manuscript.

Field-specific reporting

Please select the one below that is the best fit for your research. If you are not sure, read the appropriate sections before making your selection.

☐ Life sciences ☐ Behavioural & social sciences ☒ Ecological, evolutionary & environmental sciences

For a reference copy of the document with all sections, see nature.com/documents/nr-reporting-summary-flat.pdf

Ecological, evolutionary & environmental sciences study design

All studies must disclose on these points even when the disclosure is negative.

Study description	We calculated speciation rates using two approaches -- across a phylogeny, and from fossil occurrence data in a Bayesian framework -- and then asked whether speciation rates correlate with diversity. We did the same for extinction within the Bayesian framework.
Research sample	All hominin species
Sampling strategy	N/A
Data collection	All data were taken from previous publications (i.e., fossil occurrence data, first appearance date data, phylogeny).
Timing and spatial scale	N/A
Data exclusions	No data were excluded from the study.
Reproducibility	N/A
Randomization	N/A
Blinding	N/A

Did the study involve field work? ☐ Yes ☒ No

Reporting for specific materials, systems and methods

We require information from authors about some types of materials, experimental systems and methods used in many studies. Here, indicate whether each material, system or method listed is relevant to your study. If you are not sure if a list item applies to your research, read the appropriate section before selecting a response.

Materials & experimental systems

n/a	Involved in the study
☒	☐ Antibodies
☒	☐ Eukaryotic cell lines
☐	☒ Palaeontology and archaeology
☒	☐ Animals and other organisms
☒	☐ Clinical data
☒	☐ Dual use research of concern
☒	☐ Plants

Methods

n/a	Involved in the study
☒	☐ ChIP-seq
☒	☐ Flow cytometry
☒	☐ MRI-based neuroimaging

Palaeontology and Archaeology

Specimen provenance	N/A - all data comes from previous publications
Specimen deposition	N/A
Dating methods	N/A

☐ Tick this box to confirm that the raw and calibrated dates are available in the paper or in Supplementary Information.

Ethics oversight	N/A

Note that full information on the approval of the study protocol must also be provided in the manuscript.
